# Bioengineered Bovine Papillomavirus L1 Protein Virus-like Particle (VLP) Vaccines for Enhanced Induction of CD8 T Cell Responses through Cross-Priming

**DOI:** 10.3390/ijms24129851

**Published:** 2023-06-07

**Authors:** Raphael P. Viscidi, Treva Rowley, Ioannis Bossis

**Affiliations:** 1 Department of Pediatrics, Johns Hopkins University School of Medicine, Baltimore, MD 21218, USA; drowley6@jhmi.edu; 2 Department of Animal Production, School of Agricultural Sciences, Forestry & Natural Resources, Aristotle University of Thessaloniki, 54124 Thessaloniki, Greece

**Keywords:** virus-like particle (VLP), papillomavirus, vaccine, cross-priming, cancer vaccine

## Abstract

Safe and effective T cell vaccines are needed for the treatment or prevention of cancers as well as infectious agents where vaccines for neutralizing antibodies have performed poorly. Recent research highlights an important role for tissue-resident memory T cells (T_RM_ cells) in protective immunity and the role of a subset of dendritic cells that are capable of cross-priming for the induction of T_RM_ cells. However, efficient vaccine technologies that operate through cross-priming and induce robust CD8+ T cell responses are lacking. We developed a platform technology by genetically engineering the bovine papillomavirus L1 major capsid protein to insert a polyglutamic acid/cysteine motif in place of wild-type amino acids in the HI loop. Virus-like particles (VLPs) are formed by self-assembly in insect cells infected with a recombinant baculovirus. Polyarginine/cysteine-tagged antigens are linked to the VLP by a reversible disulfide bond. The VLP possesses self-adjuvanting properties due to the immunostimulatory activity of papillomavirus VLPs. Polyionic VLP vaccines induce robust CD8+ T cell responses in peripheral blood and tumor tissues. A prostate cancer polyionic VLP vaccine was more efficacious than other vaccines and immunotherapies for the treatment of prostate cancer in a physiologically relevant murine model and successfully treated more advanced diseases than the less efficacious technologies. The immunogenicity of polyionic VLP vaccines is dependent on particle size, reversible linkage of the antigen to the VLP, and an interferon type 1 and Toll-like receptor (TLR)3/7-dependent mechanism.

## 1. Introduction

The development of effective and safe T cell vaccines has lagged significantly behind that of vaccines for generating neutralizing antibodies. The principal cellular effector mechanism of adaptive immunity against intracellular pathogens and malignant cells is cytotoxic T cells (CTLs), also referred to as CD8+ T cells. The generation of CTLs is a complex process, but one key step is the presentation of antigen to form a peptide MHC class I complex, which is the ligand for a CD8+ T cell receptor. The formation of peptide MHC class I complexes from antigens expressed in the cytosol is referred to as classical or direct antigen presentation. Most current technologies for T cell vaccines against infectious agents and cancer have exploited this direct pathway by delivering a gene or mRNA for an antigen into an antigen presenting cell (APC) as either plasmid DNA, a recombinant viral vector, or mRNA. To date, these technologies, especially when different platforms are used in heterologous prime boost regiments, have been the most potent means to induce circulating T cells. However, the track record for success of these technologies in clinical trials is poor. Plasmid DNA is poorly immunogenic in humans [1], and lingering concerns over safety persist [2]. Viral vectors induce robust T cell responses, but pre-existing immunity in human populations can result in unexpected adverse events, as evidenced by the STEP trial where vaccination with a type 5 adenovirus expressing human immunodeficiency virus (HIV) antigens was associated with increased susceptibility to HIV infection [3]. Another example of unexpected adverse events associated with adenovirus-based vaccine is venous thrombosis and thrombocytopenia reported after the use of this vaccine technology for COVID-19 [4,5]. While mRNA vaccines have performed well for induction of antibody responses, as evidenced by the COVID-19 vaccines, experience to date in clinical trials of cancer vaccines using mRNA technology vaccines is limited. 

An alternative to the classical, direct pathway of antigen presentation involves the endocytosis of proteins by a subset of dendritic cells (DC), which are the most potent APCs. These cells process and present antigens to CD8+ T cells by cross-priming [6,7]. Cross-priming is critical to stimulate CTL-mediated immunity by subunit-protein-based vaccines. Whether there are significant biological differences in cross priming versus direct presentation is unknown. However, recent studies by Salvadore Iborra and colleagues [8] have shown that cross-priming by tissue-resident CD8α+/CD103+ DCs, also named cDC1 cells, is critical to generate tissue-resident memory T cells (T_RM_ cells). Furthermore, research in immunology over the past decades has shown the importance of tissue-resident T cells for protection at sites of infection and cancer [9,10,11].

**Figure 1:**
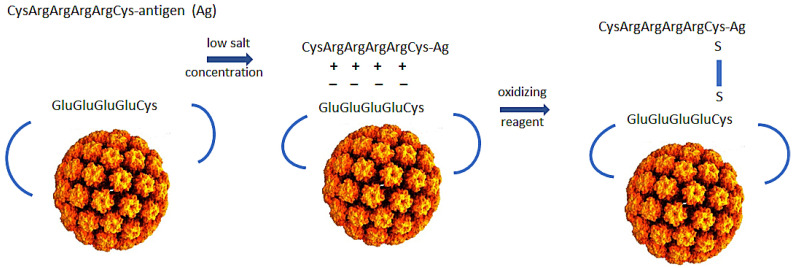
Design of polyionic bovine papillomavirus L1 protein VLP vaccine. Bovine papillomavirus L1 capsid protein with wild-type amino acids in the HI loop replaced by eight glutamic acids and a cysteine self-assembles into a VLP when expressed in insect cells from a recombinant baculovirus. To construct a vaccine, pre-reduced peptide/protein antigen is incubated with purified VLPs in a low salt buffer and oxidizing reagent to link antigen to the VLP by a disulfide bond. The charged nature of reactants inhibits homodimers (peptide-peptide or VLP-VLP) and promotes formation of the desired heterodimers between antigen and VLP.

We have designed a subunit vaccine platform that induces robust CD8+ T cell responses through the cross-priming pathway, binds and enters cDC1 cells, and has self adjuvanting activity. The property of self adjuvanting achieves two desirable goals. First, it delivers antigens and signals two and three to the same cell. Second, eliminating the need for high doses of adjuvant minimizes toxicity. Our vaccine platform consists of a genetically engineered bovine papillomavirus L1 protein that self assembles into a virus-like particle (VLP) with a surface exposed polyglutamic acid and cysteine motif that allows the linkage of polyarginine and cysteine-tagged peptide/protein antigens to the VLP (Figure 1). Due to the charged properties of the attachment site on the VLP and the tagged antigen, we designate the technology polyionic VLPs.

Herein, we describe the production of polyionic VLPs, the immunostimulatory properties of papillomavirus VLPs, the immunogenicity and efficacy of polyionic VLP vaccines in animal models, and what is known about the mechanism of polyionic VLP-induced immune responses. In this review, we have primarily included data that have been obtained in our laboratories during the last 12–13 years. In addition, we have incorporated major advances in the field in recent years that demonstrate the basic mechanisms for the efficacy of the vaccine platform. The hypotheses of this research, which is still a work in progress, are that genetically engineered papillomavirus VLPs linked to antigens by a reversible disulfide bond and polycationic amino acid sequences can induce robust CD8+ T cell responses through cross-priming, and that these VLPs demonstrate efficacy in animal models of cancer and infectious diseases by the generation of tissue-resident memory T cells. 

## 2. Production of Polyionic Bovine Papillomavirus L1 VLPs 

**Figure 2:**
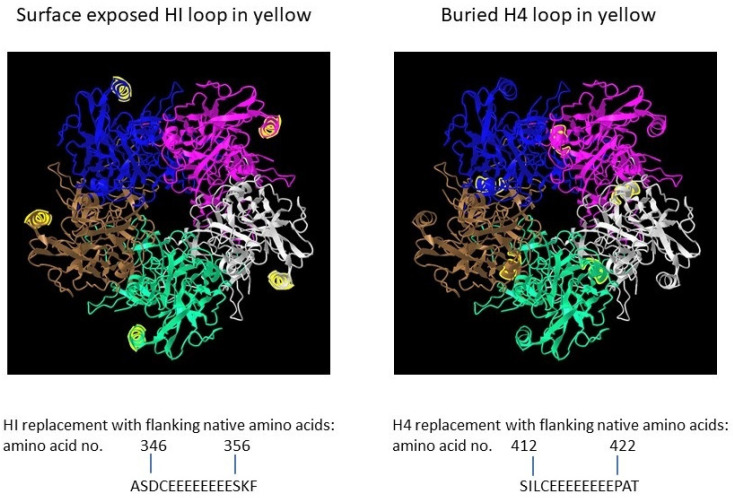
Papillomavirus pentamer highlighting the surface exposed location of the HI loop and the buried location of the H4 loops. The capsomeric structure is that of human papillomavirus type 11 L1 (PDB: 2R5K; PDB DOI: 10.2210/pdb2R5K/pdb [13]), which is closely related to that of bovine papillomavirus L1. Location numbers and flanking amino acid sequences are from capsid protein L1 of delta papillomavirus 4 (accession number: NP_056744.1). The 5 L1 proteins forming the pentamer are colored blue, magenta, white, green and brown. The HI loop region is in yellow.

The detailed production of polyionic VLPs has been previously presented by us [12]. To provide an in-depth view of the production strategy and analytical laboratory procedures, we will present below the critical steps and aspects, as well as some structure information derived from prediction based on the Protein Bank database algorithms and 3D-annotated structures. The full-length open reading frame (ORF) of bovine papillomavirus (BPV) type 1 L1 (GenBank: BBB04658.1) preceded by a Kozak consensus sequence, with codons modified for efficient expression in insect cells, was constructed by PCR-based gene synthesis. The ORF contained the insertion of a peptide with eight glutamic acid residues and a cysteine residue (E8C) and the deletion of nine wildtype amino acids in the HI loop (aa347-355 were deleted) (Figure 2). 

To evaluate effect of particle size on immunogenicity, an L1 ORF with the deletion of wild-type amino acids and the insertion of E8C in the H4 loop (aa413-421 were deleted) was also constructed [12]. The position of the H4 loop modification disrupts inter-capsomeric interactions necessary for capsid assembly. The modified BPV L1 genes were subcloned into a baculovirus transfer vector, and each transfer vector was co-transfected with linear baculovirus DNA in Spodoptera frugiperda sf9 cells. For the production of VLPs, Trichoplusia ni (High Five) cells were infected with a high-titer recombinant baculovirus stock. VLPs were purified from infected insect cells by cycles of freezing and thawing in the presence of protease inhibitors. The clarified cell lysate was extracted with an inorganic solvent (Vertrel DF) and layered over 40% sucrose. The sucrose pellet was resuspended in a high salt buffer, incubated with Salt Active Nuclease (Articzyme), and dialyzed into a storage buffer containing 0.5 M NaCl, Tween 80, carboxymethyl cellulose, and FeCl2. Analysis of the purified preparations by electron microscope confirmed that the HI-loop-engineered L1 protein formed a fully assembled VLP with an approximate size of 45–55 nm and the H4-engineered loop formed capsomeres with approximate size of 4–5 nm [12]. 

## 3. Formulation of Polyionic VLP Vaccines 

The VLP exposes, on the surface, a motif of glutamic acids and a cysteine residue that allows the linkage of antigens of a diverse length, containing a N-terminal tag of eight arginine residues with a single C-terminal cysteine residue or flanking cysteine residues. The linkage reaction involves electrostatic interactions between the charged residues of the tag (polyarginine) and VLP (polyglutamic acid) and the formation of a reversible disulfide bond by an oxidation reduction reaction. Chemically synthesized tagged peptides ranging in size from 9 amino acids to 40 amino acids have been linked to the VLP. The C terminus of the tag consists of two alanine amino acids and a tyrosine or variations thereof as a processing signal for proteases active in MHC class I presentation. Prior to the conjugation reaction, peptide antigens were reduced with Bond-breaker TCEP solution for 20 min at 50 °C. Stock VLP preparations were dialyzed into a physiological salt buffer at 1 mg/mL prior to conjugation. VLPs and peptides at peptide to L1 protein ratios between 4:1 to 8:1 were incubated overnight at 37 °C in the presence of a 5:1 ratio of glutathione disulfide (GSSG) to reduced glutathione (GSH). After the conjugation reaction, VLP vaccine preparations were dialyzed in high salt buffer containing Tween 80, carboxymethyl cellulose, and a 10:1 ratio of GSSG to GSH, aliquoted and stored at −20 °C. The amount of peptide bound to the VLP was determined by ELISA using an antigen-specific monoclonal antibody or by SDS-PAGE analysis. VLPs linked to a MUC1 peptide were analyzed with immunogold-labeled anti-MUC1 monoclonal antibody. Electron microscopy verified the integrity of the VLPs and the successful attachment of the MUC1 peptide [12]

## 4. Immunological Properties of Papillomavirus VLPs

### 4.1. Activation and Maturation of Dendritic Cells

The immunostimulatory properties of bovine and human papillomavirus VLPs have been studied by in vitro treatment of diverse myeloid cells. BPV-L1/L2 VLPs produced in insect cells were shown by confocal microscopy to bind to immature mouse bone marrow derived dendritic cells (BMDCs) and to upregulate MHC class I and II molecules, costimulatory molecules (CD40, CD80, CD86), and the CD54 adhesion molecule [14]. The requirement for a well-ordered intact capsid structure was demonstrated by the 10-fold lower effectiveness of an assembly-deficient mutant HPV16 L1 to induce phenotypic DC maturation compared to a fully assembled HPV 16 L1 VLP [15]. In addition, human polyomavirus VLPs, which share an icosahedral structure with HPV VLPs, were bound and internalized by BMDC but failed to induce the upregulation of MHC and costimulatory molecules, indicating that a similar capsid structure alone was not responsible for the immunological properties of papillomavirus VLPs. HPV16 L1 VLP-treated BMDC induced the secretion of IL-6 and TNF-a in a dose-dependent manner. When BMDCs exposed to HPV 16 L1 VLPs were co-cultured with syngeneic T cells or treated with IFN-gamma to provide additional stimulation, the cells secreted IL-12p70. In contrast, BKV VP1 and JCV VP1 polyomavirus VLPs did not induce BMDCs to secrete proinflammatory cytokines [14]. 

HPV 16 VLPs have also been shown to bind human dendritic cells derived from monocytes cultured with GM-CSF and IL-4 [14]. VLPs induced the upregulation of co-stimulatory markers and MHC molecules. Furthermore, human DCs exposed to HPV 16 VLPs expressed high levels of IL-1β and IL-12. Surprisingly, the percentage of IL-1β and IL-12producing DCs upon VLP exposure was significantly higher than that induced by LPS. The high level of expression of IL-12 was supportive of a Th1 type immune response. The ability of HPV VLPs to be taken up by human dendritic cells and induce the activation of these cells was independently confirmed in studies that showed HPV 16 L1/L2 VLPs, chimeric HPV 16 L1 VLPs, and animal papillomavirus L1 VLPs bound to the cell surface of in vitro-generated human DCs and upregulated CD80, CD86, and MHC class I and II molecules [16,17]. The uptake of VLPs was inhibited by cytochalasin D, indicating an active-process-like endocytosis. Human DCs incubated with HPV 16 VLPs were also shown to secrete IL-12 (p70) into the supernatant, at levels equivalent to that induced by LPS. The VLP specificity of the activation was demonstrated by the failure of crude insect cell lysates or heat-denatured VLPs to activate DC.

### 4.2. Stimulation of Macrophages, Monocytes, and Plasmacytoid Dendritic Cells 

HPV VLPs have also been shown to bind other myeloid cells, including human macrophages and monocytes [14]. The exposure of monocytes to HPV16 VLPs results in significant upregulation of CD54, CD40, and CD86, without significant induction of MHC class I. Despite the binding of VLPs, these markers were not upregulated in macrophages. Monocytes showed moderate expression of IL-1β and TNFα in response to HPV16 VLPs, whereas macrophages showed a more pronounced response, with the production of IL-1β, TNFα, and IL-6 upon exposure to VLPs. The cytokine response observed upon VLP exposure suggested that monocytes and macrophages may have contributed to the immunogenicity of VLPs. HPV16 L1 VLPs have also been shown to be rapidly taken up by plasmacytoid DC (pDC) but failed to induce phenotypic maturation of the cells [18]. However, pDC incubated with HPV16 L1 VLP induced the secretion of IFN-a, TNF alpha, IL-6, and IL-8, suggesting that pDC may participate in the immunogenicity of papillomavirus VLPs.

### 4.3. Activation of T Cells by Antigen-Specific Polyionic VLPs

Dendritic cells treated with papillomavirus VLPs have been shown to activate T cells [12,15,16,18,19,20]. For example, VLP-pulsed BMDC cocultured with syngeneic, naive T cells induced significant T cell proliferation and culture supernatants contained high levels of IFNγ while lacking detectable levels of IL-4 or IL-10, demonstrating that VLP-pulsed BMDCs induce Th1-dominated immune responses in T cells [12]. When DC generated from peripheral blood lymphocytes (PBL) collected from healthy HLA-A*0201-positive donors were loaded with a HPV16-L1L2-E7 chimeric VLP and cocultured with nonadherent autologous PBL, an E7-specific response was detected by IFNγ ELISPOT, demonstrating that DCs take up and process VLP particles for presentation and induction of a specific CTL response in vitro [15]. 

## 5. Immunological Properties of Polyionic VLPs

**Figure 3:**
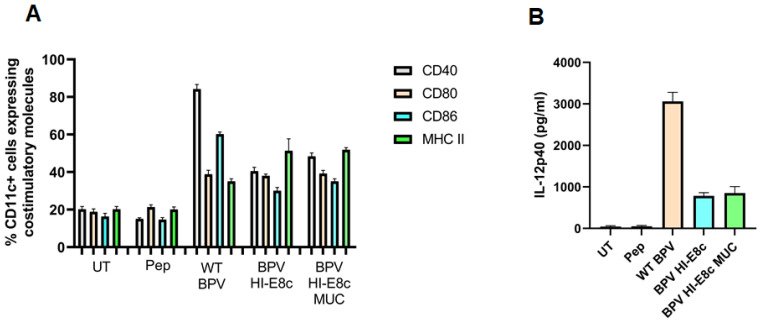
Upregulation of dendritic cell (DC) activation markers and secretion of IL-12 by bone-marrow-derived DCs treated with polyionic VLPs. (**A**) Bone marrow DCs were loaded with various BPV constructs (WT BPV, BPV-HI-E8c-MUC1, BPV-HI-E8c; BPV-H4-E8c-MUC1, BPV-H4-E8c) for 24 h, and, subsequently, were stained for standard DC maturation markers CD40, CD80, CD86, and MHC class II and analyzed by flow cytometry (BPV HI-E8c vs. untreated (UT): *p* < 0.001; BPV HI-E8c MUC1 vs. UT: *p* < 0.001). (**B**) Supernatants harvested from DC cultures, 24 h post-treatment with various constructs, were used to assess IL-12 secretion using IL-12 ELISA (BPV HI-E8c vs. UT: *p* < 0.05; BPV HI-E8c MUC1vs. UT: *p* < 0.01). pep = MUC1 peptide (250 ng-GVTSAPDTRPAPGSTAPPAH). Statistical significance was calculated by a two-tailed t-test. RAdaptedwith permission from Ref. [12] Copyright 2010 Springer Nature.

Polyionic VLPs alone and polyionic VLPs conjugated to a peptide antigen retain the immunological properties of papillomavirus VLPs [12]. BMDC exposed to polyionic VLPs (BPV-HI-E8c unconjugated) or polyionic VLPs linked to a MUC1 peptide antigen (BPV-HI-E8c-MUC1 (conjugated) and polyionic capsomeres (BPV-H4-E8c) or polyionic capsomeres linked to MUC1 (BPV-H4-E8c-MUC) significantly increased the expression of activation and maturation molecules (CD40, CD86, CD80, and MHC class II), with levels only slightly less than that induced by wild-type BPV VLPs (Figure 3A). The BPV-H4-E8c capsomeres induced an increase in some activation markers, but the response was lower than that of fully formed VLPs. BMDC exposed to antigen-conjugated or unconjugated polyionic VLPs also produced IL-12p40, but at a level lower than induced by wild-type BPV VLPs (Figure 3B). In contrast, polyionic capsomere VLPs very weakly stimulated the production of IL-12p40. To evaluate the ability of polyionic VLPs to activate T cells, splenocytes from MUC1-specific TCR transgenic mice, which provide the APC and a high frequency of naive MUC1-specific T cells, were cultured with a soluble MUC1 peptide or with MUC1-conjugated or unconjugated polyionic VLPs. Following culture with the MUC1 decorated polyionic BPV particles, but not with unconjugated polyionic BPV particles, MUC1-specific TCR transgenic splenocytes secreted significant amounts of IFNγ [12]. Polyionic capsid HI VLPs and polyionic capsomere H4 VLPs induced comparable levels of IFNγ. The responses to MUC1 conjugated to polyionic VLPs were significantly higher than induced by free MUC1 peptide.

## 6. Immunogenicity of Polyionic VLP Vaccines in Mice 

### 6.1. Immunogenicity of Polyionic VLPs Formulated with Tumor Antigens

**Figure 4:**
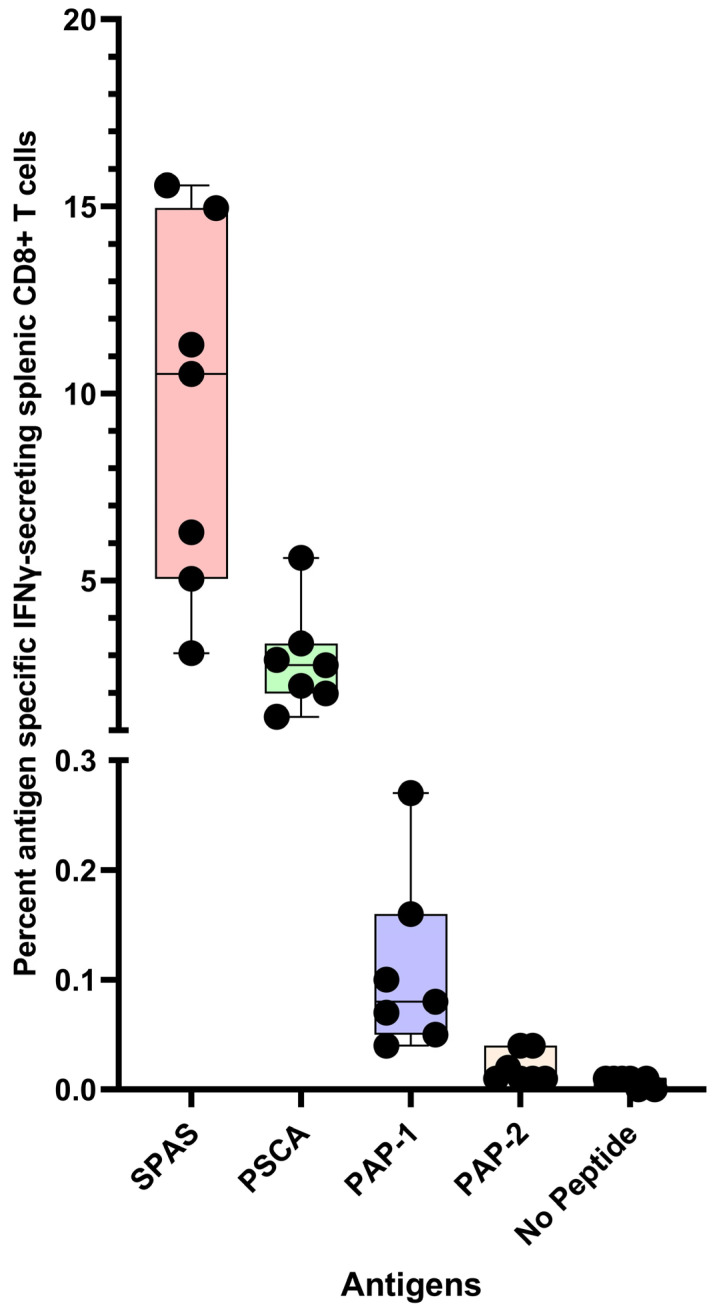
Antigen-specific CD8+ T cell response of mice to prostate tumor antigen polyionic VLP vaccination. Legend: C57BL/6 mice (*n* = 7 mice) were immunized weekly three times with 20 μg of polyionic VLP vaccine delivered by intradermal, intramuscular, and intravenous administration. Vaccines were formulated with polyarginine cysteine tagged peptides for SPAS (STHVNHLHC), PSCA (NITCCYSDL), or PAP (SAMTNLAAL and ISIWNPRIL). Frequencies of antigen-specific CD8+ splenocytes 14 days after vaccination were determined by intracellular cytokine flow assay. Compared to no peptide control, the frequency of antigen-specific CD8+ T cells was significantly increased in all groups (SPAS, *p* = 0.0002; PSCA, *p* = 0.0001; PAP1, *p* = 0.006; PAP2, *p* = 0.02, two-tailed *t*-test). Data are displayed as a minimum to maximum box plot with overlaid data points and created in GraphPad Prism 9.5. The box contains data from the 25th to 75th percentile, and the whiskers extend to the maximum and minimum values. The median is marked by a bar within the box. All individual data points are displayed. Reprinted with permission from Ref. [21] Copyright 2020 Springer Nature.

The immunogenicity of polyionic VLP vaccines was determined using vaccines formulated with self-tumor antigens. VLPs were formulated with extended peptides encoding known class I epitopes of three tumor antigens, a murine neoantigen, a stimulator of prostatic adenocarcinoma-specific T-cells-1 (SPAS), a prostate stem cell antigen (PSCA), and two peptides of murine prostatic acid phosphatase (PAP1 and PAP2). Mice on a C57BL/6 genetic background that spontaneously developed orthotopic prostate tumors (TRAMP mice, see below on Figure 4) were immunized with 20 μg of VLP protein by intramuscular, intradermal, and intravenous injections. The intramuscular dose was given into the thigh muscle. The intradermal dose was injected in split doses into the skin of the back of shaved mice. The four vaccines were administered at separate sites. The intravenous dose was administered via the tail vein. Wild-type C57BL/6 mice were immunized in the same manner [21]. Fourteen-week-old tumor-bearing mice mounted a robust response to PSCA and SPAS, with mean frequencies of IFN-γ-secreting CD8+ T cells of 2.9% and 9.5%, respectively (Figure 4). Responses to two PAP peptides (PAP-1 and PAP-2) were weak but significantly above background levels, with mean frequencies of IFN-γ-secreting CD8+ T cells of 0.11% and 0.02%, respectively. Wild-type mice and tumor-bearing mice mounted comparable responses to immunization. For some vaccines tested in TRAMP mice, the immunogenicity was lower in TRAMP mice than in wild-type mice and has been interpreted as possible systemic immunosuppression in tumor-bearing mice [22,23]. The immunogenicity of polyionic VLPs was not impaired by the tumor-bearing state. 

### 6.2. Immunogenicity of Polyionic VLPs Formulated with Microbial Antigens 

Polyionic VLP vaccines have also been formulated with antigens from several infectious agents. A vaccine was formulated with the SYVPSAEQI CD8 epitope of P. yoelii. To assess immunogenicity, a low number (3000 per mouse) of SYVPSAEQI-specific TCR-Tg CD8 T cells were transferred to normal Balb/c mice. One day later the mice were immunized intradermally (i.d.) or intravenously (i.v.) with the polyionic VLP vaccine. Expansion of TCR-Tg CD8+ T cells was analyzed at day 12 post-immunization. Mean frequencies of TCR-Tg cells to total CD8+ T cells were 0.44% and 2.1% in i.v. and i.d. immunized mice, respectively. Although i.v. immunization with polyionic VLP induced a lower level of expansion compared to i.d. immunization, it elicited a higher proportion of Ag-specific TCR-Tg CD8 T cells expressing CD69 (an early activation marker) and KLRG-1 (a marker for terminal effectors and replicative senescence). Whether this is a common feature of T cell repossess induced by polyionic VLP vaccines or whether it is a correlate of efficacy is unknown. A polyionic VLP vaccine was also formulated with a C57BL/6-restricted class I epitope and flanking amino acids from the dengue virus NS4B protein (IGCYSQVNPITLTAA). Mice (*n* = 5) were immunized intradermally with 25 μg of VLP vaccine twice 2 weeks apart. The polyionic VLP vaccine induced a mean frequency of antigen-specific CD8+ T cells of 3.2% of total CD8+ splenocytes.

## 7. Comparison of Immunogenicity of Polyionic VLPs and Other Vaccine Platforms 

### 7.1. Prostate Tumor Antigens 

Comparison of immunogenicity across vaccine platforms is complicated by differences in assays to measure antigen-specific T cell responses and the lack of standardization of these assays. No other vaccine technologies have been formulated with the SPAS1 neoantigen of TRAMP mice, and only one other research group has constructed a vaccine formulated with the prostate stem cell antigen (PSCA) [24]. In that study, the immunization of mice with a DNA vaccine prime and a Venezuelan Equine Encephalitis virus self-amplifying mRNA vaccine boost resulted in a mean frequency of ~240 spot (ELISPOT)-forming cells per 106 splenocytes. This response was much lower than the 2.9% antigen-specific CD8+ T cell response to polyionic VLP vaccination (see above), assuming that 240 spots per million splenocytes corresponded to ~0.3% antigen-specific CD8+ T cells by intracellular cytokine stain (ICS) assay (based on assumption that 25% of splenocytes are CD3+ T cells, and one-third of these cells are CD8+). In a similar study, a comparable prostate tumor antigen, STEAP1, generated an antigen-specific CD8+ T cell response of 1.14% by ICS assay following immunization with a recombinant Chimpanzee adenovirus prime and vaccinia boost [22]. 

### 7.2. HPV 16 E7 Antigens

Historically, HPV 16 E7 is one of the most studied tumor antigens. In a comparative study of DNA vaccine constructs formulated with HPV 16 E7, the most potent construct was a plasmid encoding a calreticulin (CRT)-E7 fusion protein. Immunization of mice with this construct generated ~924 antigen-specific IFNγ-secreting CD8+ T cells per 3 × 105 splenocytes, which corresponded to an approximately 3.7% frequency of antigen-specific CD8+ T cells [25]. In a BioNTech SE study of an mRNA vaccine encoding the HPV 16 E7 protein, a 4% frequency of E7-specific splenic CD8+ T cells was reported [26]. E7 peptide antigens have also been delivered with nanoparticles. A hydrophilic polyester nanoparticle loaded with E7 peptide and adjuvanted with poly I:C induced a 1% frequency of E7 specific CD8+ T cells [27]. A 30nm polymeric nanoparticle with a surface exposed cysteine residue was formulated with a thiol containing E7 peptide and administered with a CpG adjuvant. The vaccine induced a 9% frequency of E7 specific splenic T cells [28]. Of note, this nanoparticle used a reversible disulfide bond for attachment of antigen to the VLP. However, unlike polyionic VLPs, an exogenous adjuvant (CpG) had to be used in order to induce an immune response. CpG poses a potential risk for adverse events in human studies. 

**Figure 5:**
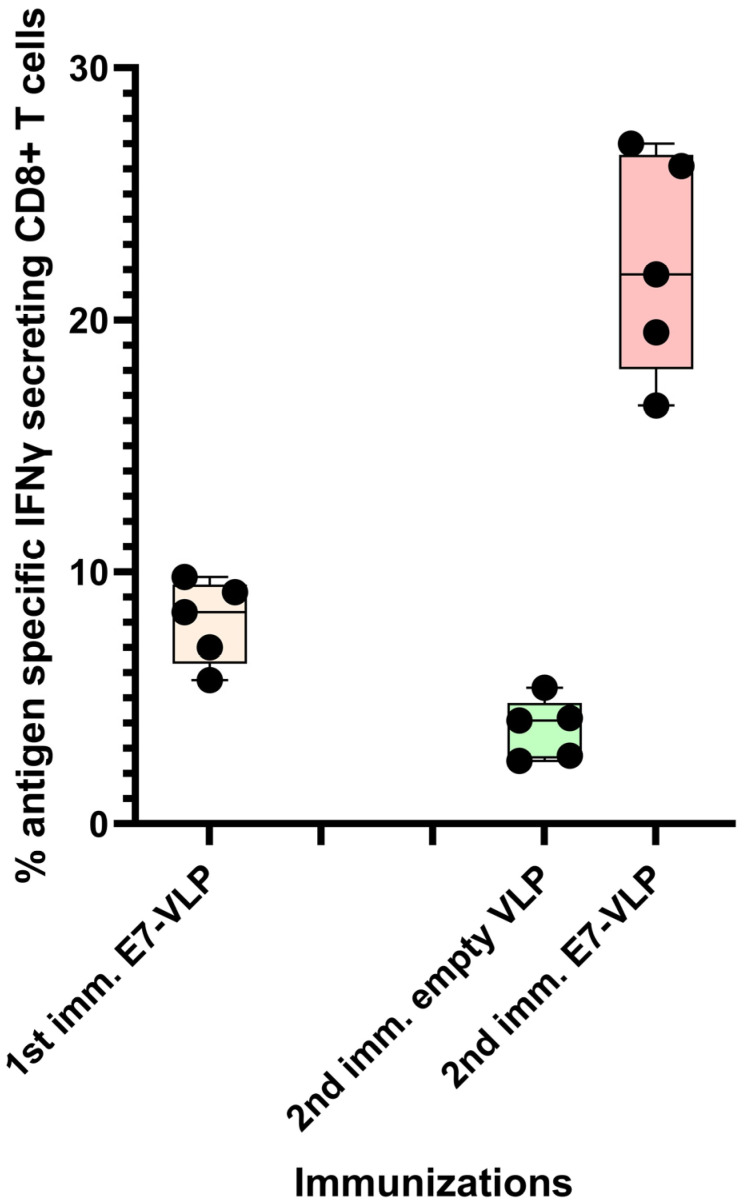
Primary immunization with polyionic E7 VLP vaccine and repeated immunization after 3 months. In total, 15 C57BL6 mice were immunized weekly three times × by intradermal injection with a polyionic VLP vaccine formulated with HPV type 16 H-2Db-restricted E7 epitope (E749-57; RAHYNIVTF). In total, 5 mice were euthanized 10 days after the last dose of vaccine, and the frequency of splenic antigen-specific CD8+ T cell was measured by ICS assay. After 3 months, 5 mice were reimmunized with the polyionic E7 VLP vaccine, and another 5 mice were immunized with an empty polyionic VLP as a control. Mice were euthanized 10 days after the last dose of vaccine to measure splenic antigen-specific CD8+ T cells. The response to reimmunization was significantly higher than that after to primary immunization (Mann–Whitney test, *p* = 0.008), whereas the response after re-immunization with empty VLPs was significantly lower, consistent with contraction of the CD8+ T cell response over time. Data are displayed as described in the legend to Figure 4.

It has been our experience that the CD8+ T cell response to immunization with polyionic HPV 16 E7 peptide VLPs can be ~8%, and, after a second immunization 3 months following the first, the response can be up to 22% antigen-specific CD8+ T cells (Figure 5). Collectively, these studies demonstrated that the immunogenicity of polyionic VLPs was superior to, or as good as, other vaccine platforms formulated with the same antigen.

## 8. Application of Other VLP Platforms Technologies against Tumor Antigens

Despite the large number of experimental cancer vaccines under development, few therapeutic VLP vaccines have been formulated with tumor antigens. Technologies based on synthetic nanoparticles or long synthetic peptides delivered with adjuvants are the preferred strategies. Bacteriophage VLPs displaying HER2 or xCT proteins have been developed for the treatment of breast cancer and have shown some efficacy in animal models [29,30,31]. However, the vaccines induced antibody responses and not T cell responses. Enveloped simian immunodeficiency virus VLPs expressing mesothelin or Trop2 glycoproteins have been developed for treatment of pancreatic cancer [32,33]. The vaccines reduced tumor growth in an animal model. The vaccines induced humoral and cellular immunity and decreased Treg cells and myeloid suppressor cells in the tumor microenvironment. The mechanism of the anti-tumor effect was unclear. An empty (no antigen) cow pea mosaic virus VLP suppressed tumor growth in a melanoma model. The beneficial effect was associated with neutrophils and secretion of IL-12 and IFNγ [34]. A bacteriophage VLP with TLR9 ligand activity was formulated with various cytotoxic T cell epitopes. The vaccine inhibited tumor progression in a lung melanoma model and was shown to modestly increase CD8+ T cell numbers [35]. None of the above VLPs were similar in design to polyionic VLPs, and most have not induced robust CD8+ T cell responses. 

## 9. Immunogenicity of Polyionic VLP Vaccines in Non-Human Primates

**Figure 6:**
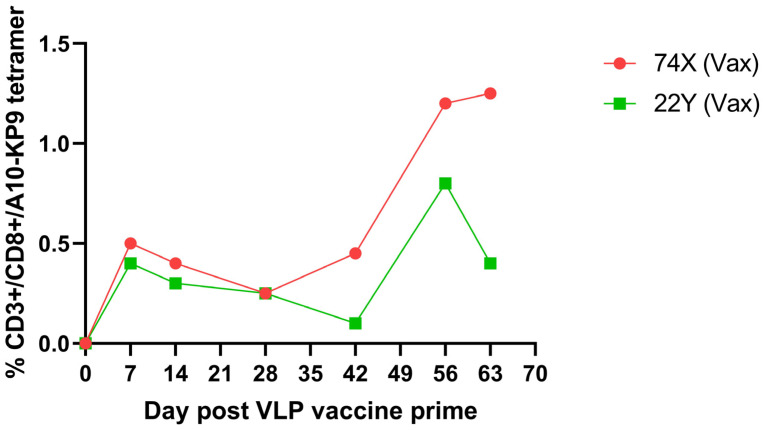
Kinetics and magnitude of K9P tetramer response to K9P-polyionic VLP immunization of two pigtail macaques. Two macaques were immunized intradermally with a VLP vaccine formulated with polyarginine-cysteine tagged KP9 peptide (KKFGAEVVP), followed by four combined intradermal and intramuscular boosts every two weeks. Blood was collected at serial time points, and the frequency of antigen-specific CD8+ T cell responses determined by flow cytometry tetramer stainingAdapted with permission from Ref. [36] Copyright 2020 Springer Nature.

Two pigtail macaques (Macaca Nemestrina) were immunized as part of a study of the potential loss of viral fitness due to a CTL escape mutation from a simian immunodeficiency virus (SIV) Gag K165R epitope, known as KP9 [36]. The macaques were immunized intradermally with a polyionic VLP vaccine formulated with the KP9 peptide, followed by four combined intradermal and intramuscular boosts every two weeks. Although responses fluctuated over time, polyionic VLP vaccination induced a strong antigen-specific CD8+ T cell response in both animals, a maximum response of 0.62% and 0.98% tetramer positive CD3+/CD8+ lymphocytes (Figure 6). 

**Figure 7:**
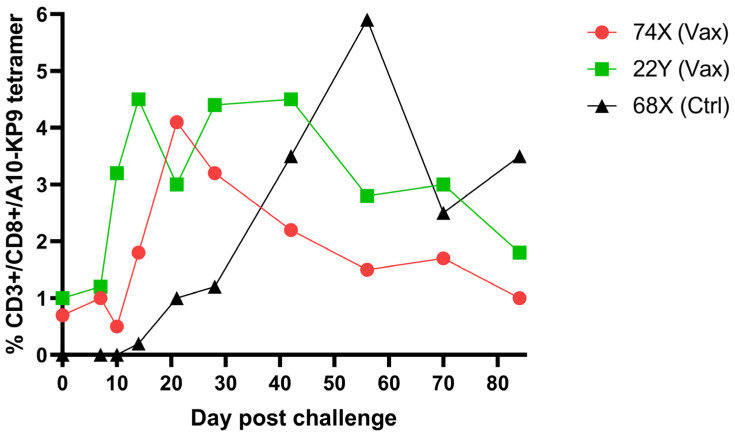
Kinetics and magnitude of K9P tetramer response in K9P-polyionic-VLP-vaccinated pigtail macaques post SIV challenge and K9P tetramer response to challenge of an unimmunized pigtail macaques. Legend: Two pigtail macaques (74X and 22Y) were immunized (Vax) as described in the legend to Figure 6, and these macaques and an unimmunized control (Ctrl) macaque (68X) were challenged intravenously with a highly virulent molecular clone of simian immunodeficiency virus, SIV/17E-Fr. Serial blood samples were collected before and after challenge. The frequency of antigen-specific CD8+ T cell responses was determined by Flow cytometry tetramer staining. Adapted with permission from Ref. [36] Copyright 2016 Springer Nature.

Not unexpectedly, a single epitope vaccine did not prevent infection with a highly virulent molecular clone SIV/17E-Fr. However, both animals responded to acute infection with an early and robust KP9-tetramer specific anamnestic response that appeared more rapidly than the response in unvaccinated animals, supporting the ability of the polyionic VLP vaccine to induce memory T cells (Figure 7). Memory phenotype analysis of tetramer positive cells from vaccinated macaques showed that 42% were transitional effector memory cells (CD95+/CD28+/CCR7−), 55% were fully differentiated effector memory cells (CD95+/CD28−/CCR7−), and 3% were central memory cells (CD95+/CD28+/CCR7+). In rhesus macaques, vaccines that induced robust effector memory T cell responses were believed to provide better protection against SIV infection than vaccines that produced predominantly central memory T cell responses [37].

## 10. Efficacy of MUC1 Polyionic VLP Vaccine in a Murine Cancer Model

**Figure 8:**
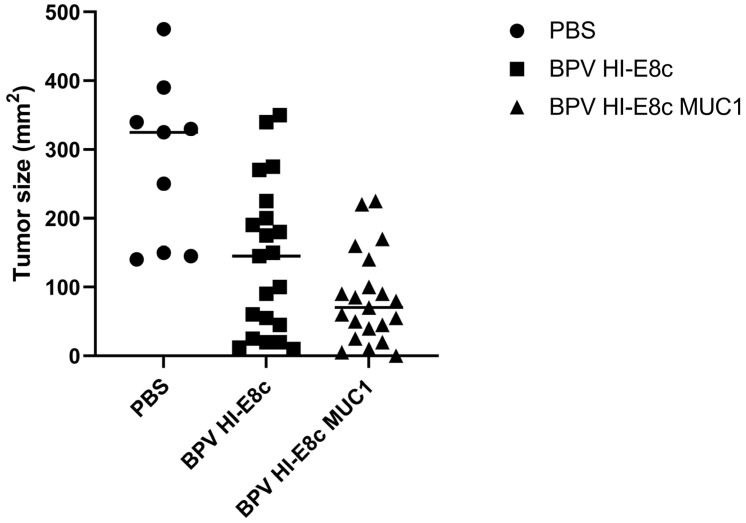
Efficacy of MUC1 polyionic VLP vaccine in murine tumor model. MUC-1 transgenic mice were immunized subcutaneously three times, 2 weeks apart with 5 μg per dose with vector alone (BPV-HI-E8c), polyionic VLP vaccine (BPV-HI-E8c-MUC1), or PBS (controls). Two weeks following the last dose of vaccine, mice were injected subcutaneously with 5 × 104 RMA-MUC1 tumor cells. Tumor growth was measured every 3–4 days using calipers On day 21, the day before the first control mouse had to be euthanized, tumors in all mice in all groups were measured. *n* = 21 per group for vaccine and vector group, and *n* = 9 mice per group for PBS group. Tumors in vector only treated mice were significantly smaller than untreated mice (*p* < 0.01). Tumors in polyionic MUC1 vaccine treated mice were significantly smaller than untreated mice (*p* < 0.001) and less than vector only treated mice (*p* < 0.05). Significance was calculated by two-tailed t-test. Adapted with permission from Ref. [12] Copyright 2010 Springer Nature.

MUC1-transgenic mice, which contain human MUC1 that is spatially and temporally expressed similarly to that in humans, were immunized subcutaneously and boosted 2 weeks later with 5 μg of MUC1 polyionic VLP vaccine (BPV-HI-E8c-MUC1) or empty vector (BPV-HI-E8c) or PBS as an untreated control. Two weeks after the last dose of vaccine, mice were challenged subcutaneously with RMA-MUC1 cells, a lymphoma T cell line that expresses MUC1. Tumor growth was monitored with calipers every 2–3 days up to 60 days. By day 30, 100% of PBS treated control mice were euthanized because their tumors reached a size of 2 cm. In contrast, mice immunized with the BPV-HI-E8c polyionic VLP vector control or the BPV-HI-E8c-MUC1 polyionic VLP vaccine showed slower growth kinetics, with a strikingly longer time to appearance of tumors in BPV-HI-E8c-MUC1-vaccinated animals Figure 8). Tumor size was measured in all mice on the day the untreated group had to be euthanized. Both empty polyionic VLP- and polyionic MUC1 VLP-treated mice had significantly smaller tumors, demonstrating the inherent anti-tumor activity of immunostimulatory polyionic VLPs. However, BPV-HI-E8c-MUC1-vaccinated mice had significantly smaller tumors than empty-vector-treated mice (Figure 8). 

## 11. Efficacy of Prostate Cancer Polyionic VLP Vaccine in a Physiologically Relevant Murine Prostate Cancer Model

### 11.1. The TRAMP Mouse Model of Prostate Cancer

As subcutaneous tumor models are not physiological for solid tumors arising in internal organs, polyionic VLP vaccine efficacy was evaluated in the transgenic adenocarcinoma of the mouse prostate (TRAMP) model that closely mirrored the pathogenesis of human prostate cancer [38]. Male TRAMP mice uniformly and spontaneously developed autochthonous (orthotopic) prostate tumors following the onset of puberty. The mice developed mild hyperplasia, followed by frank neoplasia corresponding to prostatic intraepithelial neoplasia (PIN) in men, and, eventually, well-differentiated adenocarcinoma [39,40]. One-third of animals developed anaplastic and highly invasive neuroendocrine carcinomas with a propensity for metastasis to the lungs, lymph nodes, and bones. The model was challenging for cancer therapies because tumors arise spontaneously and asynchronously from normal prostate cells by transcriptional activation of the SV40 oncogene. Growth of tumors was highly variable across individual mice, necessitating larger sample sizes than models where fixed doses of cultured tumor cells with predictable growth kinetics were injected subcutaneously. Without the removal of the prostate gland, tumor growth could be controlled, but tumors could not be fully eradicated. This situation may have been analogous to the presence of cancer stem cells in human tumors, including human prostate cancer [41,42]. The TRAMP model is not widely appreciated and uncommonly used, in part because studies of advanced stages of cancer require nearly a year to execute. 

### 11.2. Efficacy of Polyionic VLP Vaccination of Advanced Stage Cancer in TRAMP Mice

To mimic more closely the application of a prostate cancer vaccine in men, polyionic VLP vaccination was tested at 19–20 weeks of age, when many mice had adenocarcinoma [21]. Polyionic VLP vaccines were formulated with peptides encoding MHC class I-Kb or Db-restricted epitopes from PSCA, murine prostatic acid phosphatase (PAP), or SPAS. Each peptide had an N-terminal tag composed of eight arginine amino acids flanked by cysteines and followed by two alanine amino acids and a tyrosine (AAY). TRAMP mice 19–20 weeks of age were immunized three times weekly by intradermal, intramuscular, and intravenous injections. One group of mice also received three weekly intraperitoneal injections of anti-mouse PD-1 (CD279), clone RPM1-14 refer to the hybridoma cell line producing anti-mouse PD1. 

**Figure 9:**
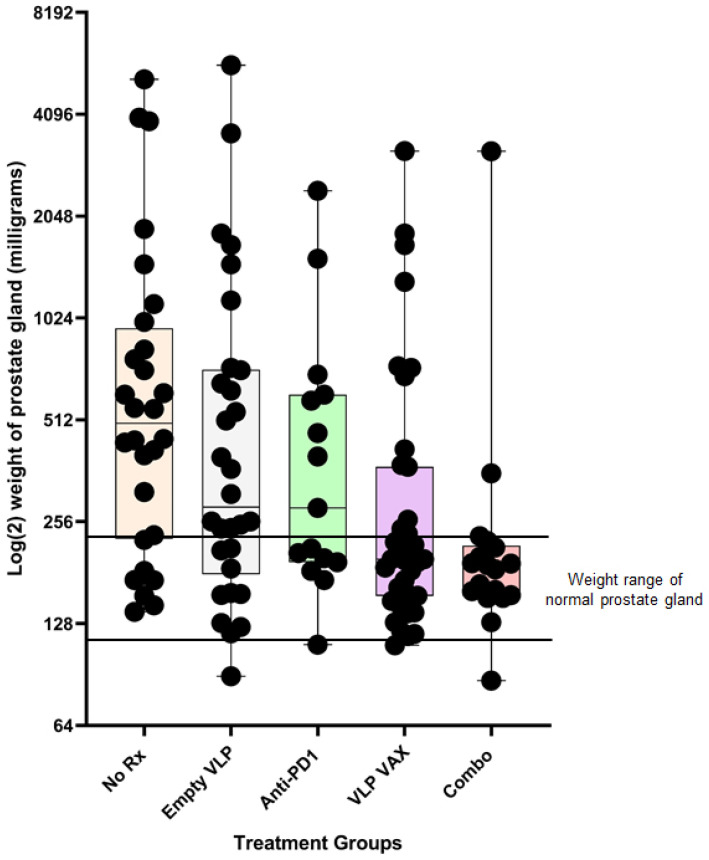
Efficacy of prostate cancer polyionic VLP vaccine in TRAMP mice. Twenty-week-old TRAMP mice were immunized weekly three times intradermally, intramuscularly, and intravenously with 20 μg of polyionic VLPs formulated with polyarginine cysteine tagged tumor antigen peptides (SPAS, PSCA, and PAP) (*n* = 39 mice), treated by intraperitoneal injection with anti-PD-1 weekly three times beginning with the second dose of vaccine (*n* = 15 mice), or treated with both VLP vaccine and anti-PD1 (Combo) (*n* = 18 mice). Mice were also treated with unconjugated polyionic VLPs (empty VLP) (*n* = 30 mice). Control mice were untreated (No Rx) (*n* = 28 mice). At 26 weeks of age, mice were sacrificed, and the dissected prostate gland was weighed. Weights are in milligrams on a Log (2) scale. The upper and lower limits of the range for the weight of a normal prostate gland (125–250 mg) are marked by horizontal lines. Data are displayed as described in the legend to Figure 4. Reprinted with permission from Ref. [21] Copyright 2020 Springer Nature.

Mice were euthanized 3 weeks after the last dose of vaccine to assess vaccine efficacy based on weight of the prostate gland (Figure 9). The normal mouse prostate gland is between 0.125 and 0.225 g. The median weight of the prostate gland of untreated mice was 0.500 g, with 79% of mice having enlarged glands due to tumor growth. Weights of untreated mice were compared to other groups by a one-tailed Mann–Whitney test. The median weight of the prostate gland of vaccine-treated mice was 0.198 g, leaving only 36% of mice with an enlarged gland (*p* = 0.0006 vs. untreated mice). The median prostate gland weight of mice treated with vaccine and anti-PD1 was 0.188 g, leaving only 17% of mice with an enlarged gland (*p* = 0.0002 vs. untreated mice). Anti-PD1 therapy alone had a modest nonsignificant effect (*p* = 0.126) on prostate weight, consistent with a previous study showing modest efficacy when the drug was administered at 12–14 weeks of age [43]. Nevertheless, the polyionic VLP vaccine in combination with anti-PD1 significantly reduced prostate weight compared to anti-PD1 therapy alone (*p* = 0.007). The empty polyionic VLP also had modest nonsignificant (*p* = 0.114) anti-tumor activity. The results may be explained by the observation that human papillomavirus VLPs directly bind to and infect tumor cell lines due to heparan sulfate proteoglycan-dependent tropism of the VLPs for disrupted epithelial and mesothelial tissues. Furthermore, papillomavirus VLPs have been shown to have anti-tumor properties in vivo [44,45,46]. An additional factor is the ability, noted above, of human papillomavirus VLPs to induce the secretion of type I interferons, which are known to have anti-tumor activity [47]. Thus, some efficacy from inherent anti-tumor and proinflammatory activities of papillomavirus VLPs was to be expected. However, the prostate cancer polyionic VLP vaccine significantly reduced prostate weight compared to empty VLPs (*p* = 0.02). 

### 11.3. Generation of Tissue Infiltrating CD8+ T Cells in the TRAMP Model

**Figure 10:**
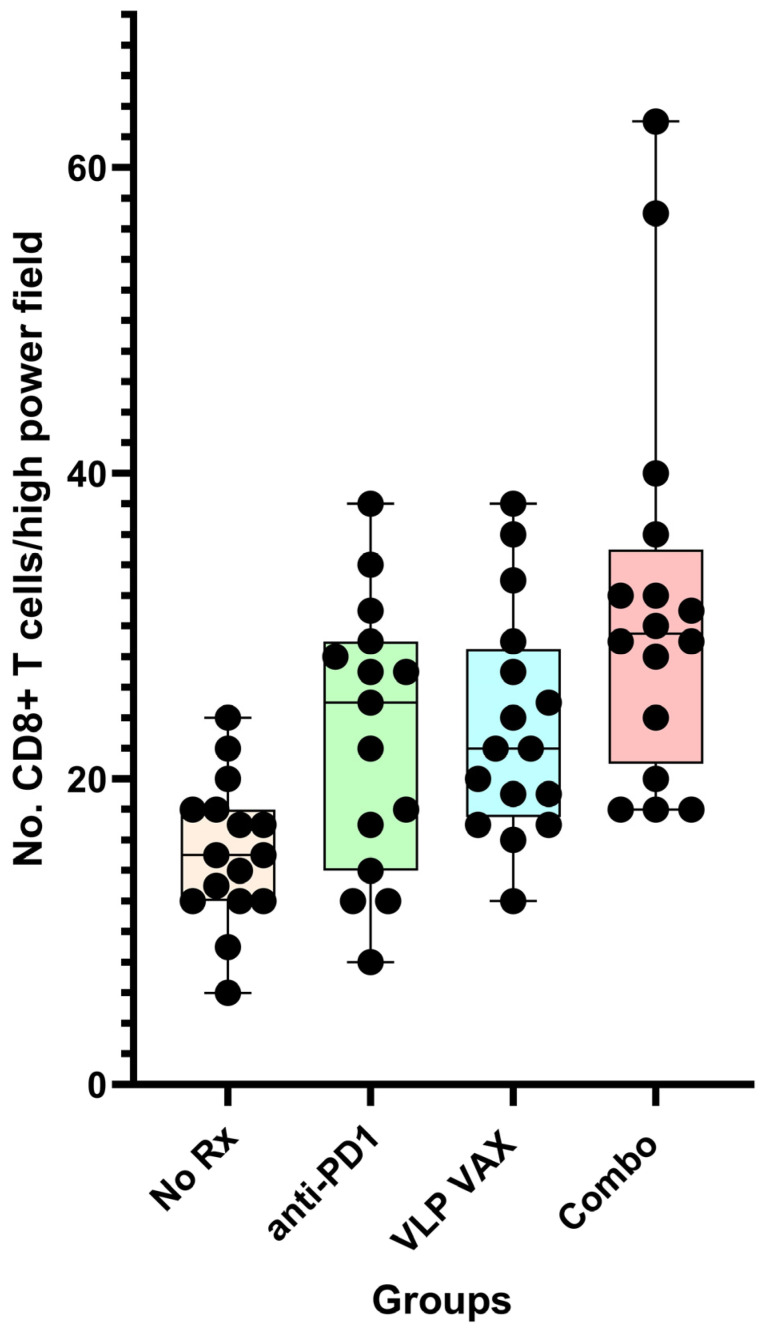
Tissue infiltrating CD8+ T cells in prostate cancer tissues of TRAMP mice. Twenty-week-old mice were treated as described in Figure 11. At 26 weeks of age, prostate tumor tissue sections were stained for CD8+ T cells. Data for CD8+ cell number are from the average of four images per mouse, with group sizes of 16 mice (15 for anti-PD1 alone). Data are displayed as described in Figure 4. Reprinted with permission from Ref. [21] Copyright 2020 Springer Nature.

Tumor tissue was evaluated by immunohistochemistry for the number of CD8+ tumor infiltrating lymphocytes (TIL) per high powered field (hpf). The median number of TILs was significantly less in untreated mice (15/hpf) compared to mice treated with vaccine (24/hpf) or vaccine + anti-PD1 (32/hpf) (*p* < 0.01) (Figure 10). Anti-PD1 treatment resulted in a frequency of TILs comparable to vaccine alone, despite the lack of anti-tumor efficacy. Studies using a subcutaneous tumor model with TRAMP C1 cells provided a potential explanation for this discrepancy between TIL numbers and efficacy. Tumor tissue was harvested, and antigen specificity of infiltrating CD8+ cells was determined by flow cytometry. The frequency of SPAS-antigen-specific CD8+ T cells was four-fold higher (~10%) in tumor tissue from polyionic VLP-immunized mice compared to mice that received vaccine and anti-PD1 (~2.5%). The finding suggested that anti-PD1 promoted the infiltration of non-antigen-specific CD8 cells. In addition to antigen specificity, the T cells recruited to the tumor site by vaccination or anti-PD1 treatment may have differed in other properties since recent studies using RNA-seq have shown considerable heterogeneity among tissue-resident T cells [48]. 

### 11.4. Comparative Studies of Efficacy of Vaccine Platforms in TRAMP Mice

The predictive value for efficacy in humans of animal models using cutaneous tumor cell grafts is problematic, which is why we used a model that is physiologically relevant: the TRAMP model of prostate cancer. The model is underappreciated because its use is limited to prostate cancer research. Comparisons across studies of vaccines used to treat TRAMP mice is difficult due to differences in endpoints. Some studies use survival, and other studies use tumor burden but measure tumor size in different ways, including genitourinary tract (GUT) weight, GUT to body weight ratio, or magnetic resonance imaging (MRI). We used prostate weight because the tumors are confined to the prostate gland. However, greater skill in anatomic dissection is needed to isolate the prostate gland away from the bulk of the total genitourinary tract tissue. 

A brief review of other vaccine studies reveals that polyionic VLPs demonstrate superior efficacy (Table 1). The vaccines tested included recombinant viral vectors that are considered the most potent platforms for induction of CD8+ T cell responses as well as plasmid DNA and self-replicating mRNA vaccines. 

A dendritic-cell-based vaccine administered at 8 weeks of age reduced the genitourinary tract (GUT) weight to body weight ratio ~33% at 28 weeks of age [49]. A simian adenovirus STEAP1 prostate tumor vaccine administered at 6–8 weeks of age, followed by a modified vaccinia Ankara (MVA) boost at 9–11 weeks of age, reduced GUT to body weight ratio by ~20% [22]. A tumor cell lysate vaccine administered in combination with anti-CTLA4 at 14 weeks of age failed to reduce prostate weight at 19 weeks of age, but the incidence of tumors (histologic invasive adenocarcinoma) was reduced from 69% in controls to 43% in the treatment group [43]. Anti-CTLA4 alone had a small anti-tumor effect at 3 weeks but not at 5 weeks post-therapy. A DNA prime MVA boost vaccine expressing PSCA and STEAP1, administered at 7 and 11 weeks of age, reduced GUT weight by ~40% at 24 weeks of age [50]. The treatment increased CD3 T cell infiltration by ~25% compared to an increase in CD8+ T cell infiltration by >50% by polyionic VLP vaccination. A tumor cell lysate vaccine co-encapsulated with CpG oligonucleotides in a poly lactide/glycolide microsphere (MS), administered at 10, 12, 14 and 16 weeks of age, reduced prostate tumor volume measured using magnetic resonance imaging (MRI) by ~80% [51]. However, MS-encapsulated CpG and poly I:C without tumor lysate reduced tumor volume by 60%. A DNA prime and Venezuelan Equine Encephalitis (VEE) self-replicating mRNA replicon particle boost, formulated with PSCA, prolonged survival by 20% at 200 days when administered at 7–8 weeks of age. Efficacy was 80% based on survival at 1 year [24]. The most important take away from these studies is that treatment was started in young TRAMP mice (~8–10 weeks of age) with pre-neoplastic lesions and demonstrated modest efficacy (20–40% reduction in GUT weight). The translational value of studies conducted in TRAMP mice at 8–10 weeks of age when the mice exhibit PIN-like lesions was limited because men with early-stage prostate cancer can be successfully treated surgically. In contrast, polyionic VLPs showed 60–80% reduction in prostate weight (efficacy of 43–63%) with treatment starting at 20 weeks of age, when mice have well developed adenocarcinoma.

## 12. Mechanisms of Polyionic VLP Vaccine-Induced Immunogenicity

### 12.1. Adjuvant Effect

The activation of an immune response requires three signals. Antigen presentation is signal one. To confer the cytotoxic effector function to CTLs also requires the upregulation of cell surface costimulatory molecules or signal two, and the secretion of cytokines, such as IL-12, is known as signal three. Signal three directly contributes to T cell differentiation and expansion. Signals two and three are typically provided by adjuvants. An adjuvant is a substance that is added to a vaccine to stimulate and enhance the magnitude and durability of the immune response. Although it is well established that adjuvants enhance antibody responses to vaccination in humans, very few adjuvants used in licensed vaccines are known to elicit strong CTL responses. Adjuvants that show modest efficacy for eliciting CD8+ T cell responses include squalene derivatives (e.g., MF59), saponin-based adjuvants (e.g., QS-21), and carbomer-based nano-emulsion (Adjuplex) (see [52] for review). These adjuvants primarily act as antigen delivery systems and perform poorly in enlarging the magnitude of CD8+ T cell memory. The development of adjuvants that induce strong cell-mediated immune responses to subunit vaccines remains a challenge, and the development of adjuvants that generate T_RM_ cells is also a challenge.

The mechanism of the adjuvant effect induced by polyionic VLPs is still poorly understood. The ability of papillomavirus VLPs produced in insect cells from recombinant baculoviruses to induce activation and maturation of dendritic cells, as described above, provides signal two, an essential step in the induction of an immune response. The ability of polyionic VLPs to induce secretion of cytokines contributes to signal three, and, specifically, the production of IL-12 and type I interferons is indicative of the ability to induce a Th1 type cellular immune response. The ability of papillomavirus VLP to induce secretion of interferon type I by plasmacytoid DC may also contribute to immunogenicity because a recent study has shown that pDCs cooperate with cDC1 cells to generate antiviral CD8+ T cell responses [53]. 

### 12.2. Particle Size and Immunogenicity

**Figure 11:**
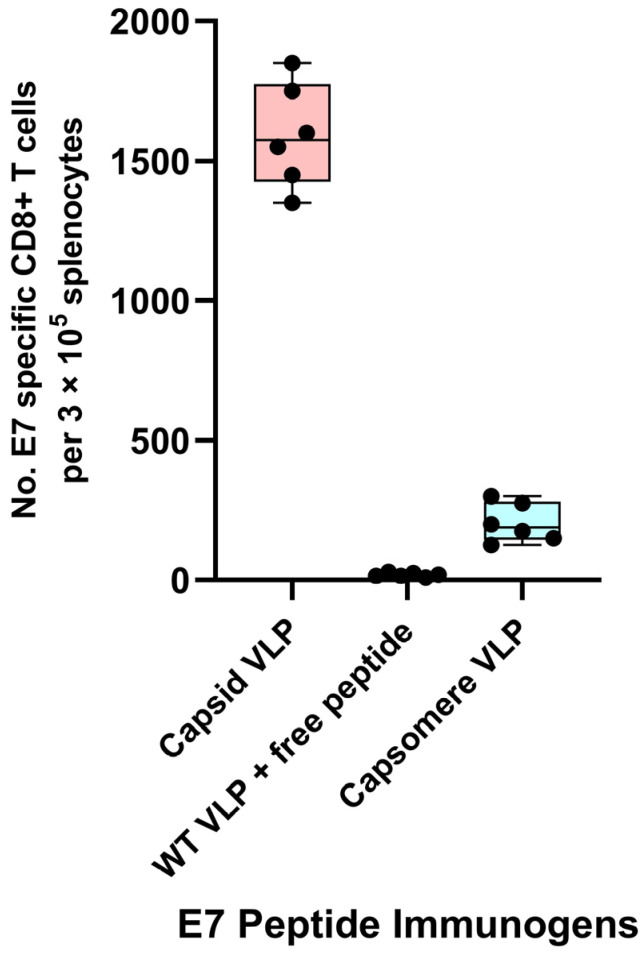
Dependence of immune response on particle size and physical linkage of antigen to the VLP. C57BL6 mice (*n* = 6 mice) were immunized subcutaneously weekly three times with 20 μg of fully assembled capsid size polyionic VLP formulated with HPV 16 E7 peptide or 20 μg of capsomere size polyionic E7 VLP, or 20 μg of unconjugated capsid size polyionic VLP mixed with free E7 peptide. Unconjugated VLPs served as a control. Ten days following the last dose of vaccine, splenocytes were stimulated with E7 peptide, CD8+ T cells were measured by Flow cytometry intracellular cytokine assay for IFNγ, and data are reported as number of positive cells per 105 splenocytes. Data are displayed as described in Figure 4.

Vaccine development in the past decades has moved away from whole-organism-based vaccines toward highly defined antigens delivered as subunit vaccines. However, peptide and protein antigens alone are not highly immunogenic. One well accepted way to improve immunogenicity is to exploit the inherent ability of the immune system to recognize small particles, such as viruses [54,55]. Thus, particulate vaccines, such as VLPs, are an attractive delivery system for peptide and protein antigens. The physical sizes and shapes of particulate vaccines have been shown to be critical determinants of immunogenicity. Polyionic VLPs are ~40–50 nm in size. Large particles (>500 nm in diameter) tend to be physically trapped at the injection site by interactions with extracellular matrix proteins, whereas ultra-small nanoparticles (<10 nm in diameter) or soluble antigen molecules can rapidly diffuse into and out of lymph nodes, thus minimizing the chance of APCs phagocytizing enough vaccine particles. On the other hand, particles of an intermediate size (10–100 nm in diameter) can both efficiently drain to regional lymph nodes and become retained there, thereby increasing the chance of antigen uptake and presentation by APCs [56,57,58]. Particle size has been shown to have a significant impact on immunogenicity. By using antigen conjugated polystyrene beads in a narrow size range between 20 to 123 nm, intradermal immunization with 40–49 nm particles was shown to enhance IFNγ-producing CD8+ T cell responses [59]. Synthetic particles 70 nm in size, compared to larger particles 300–1000 nm in size, have been shown to enhance cross-priming through the cytosolic pathway in vitro [60]. Particle shape can also influence cellular uptake. Spherical and ellipsoid structures, such as polyionic VLPs, attach to and are internalized by DCs more efficiently than rod-like structures [61]. The size-dependent immunogenicity of polyionic VLPs was compared by formulating vaccines with BPV HI VLPs (~50 nm capsids) and BPV H4 VLPs (5–10 nm capsomeres) linked to an HPV16 E7 peptide. Mice were immunized with each vaccine or a mixture of BPV HI VLPs and free peptides. The HPV 16 E7 peptide HI polyionic VLP vaccine induced a more robust CD8+ T cell response than the E7 peptide H4 polyionic VLP vaccine (Figure 11). The administration of free peptides mixed with polyionic VLPs, but not linked to the VLP by a disulfide bond, failed to induce a detectable CD8+ T cell response, demonstrating the dependence of the immune response on physical linkage of the antigen to the VLP. 

### 12.3. Reversible Linkage of Antigens to VLPs and Immunogenicity

Polyionic VLPs induce immune responses by cross-priming of exogenous antigen to generate MHC class I peptide complexes. Studies with model particles have shown that the rate of antigen release in the early endosome directly affects cross-priming efficiency, with an apparent time limit of ~25 min post phagocytosis for antigen release to be productive [62]. Cross-priming efficiency has been shown to be increased by use of a cleavable linker between the antigen and the particle. Thus, in a model system with synthetic nanoparticles, linkage of antigen to the particle by a reducible bond induced a higher frequency of CD8+ T cells than linkage of the same antigen by a non-reducible bond [63]. 

The size limit of proteins that can be linked to polyionic VLPs is unknown. However, using the same linkage technology, a protein of ~25 kDa was successfully linked to a polyionic polyomavirus VLP [64]. Importantly, for the use of polyionic VLPs as vaccines, the optimum size of the antigen will depend on both immunological and biochemical considerations. Thus, the efficiency of cross-priming is known to be inversely correlated with the length of the antigen [65]. Furthermore, the amino acid composition of the antigen is likely to influence proteolytic processing for cross-priming. 

### 12.4. Immunogenicity and Cell Penetrating Amino Acids

The polyarginine-tagged peptide likely functions as a cell-penetrating peptide once the peptide is released from the polyionic VLP by the reduced environment within endosomes. Cell-penetrating peptides (CPPs) are short diverse peptides, typically consisting of 5–30 amino acids, rich in basic or amphoteric amino acids, which display excellent abilities to penetrate various biological membranes [66]. Membrane-permeable peptides have been extensively used as carrier vectors for the intracellular delivery of various proteins and macromolecules [67]. Arginine-rich peptides are among the representative classes of these vectors. The internalization mechanism involves endocytosis and increased efficiency of translocation from the endosome into the cytosol [68]. For arginine-rich peptides, escape into the cytoplasm can be further enhanced by the proton sponge effect, whereby protonation of the guanidinium group requires chloride and water from the cytoplasm to balance charge and concentration, resulting in the expansion and rupture of the endosome and the escape of the CPP [69,70,71]. However, the utilization of CPPs is limited by a lack of selectivity, degradation by enzymes, toxic effects, reactions with plasma proteins, and inefficient escape from endosomes [72]. The delivery of polyarginine-tagged antigens with polyionic VLPs overcomes some of the limitations of CPPs by preferential uptake by dendritic cells and a “shielding and then activating” strategy [73]. Electrostatic interactions between the negatively charged polyionic VLP and CPP-tagged antigen provide the shield, and the release of the peptide within endosome by the reversal of the disulfide bond allows the activation. The importance of a CPP-tagged antigen is supported by the induction of a comparable CD8+ T cell response when the polyarginine tag is replaced by a tag composed of an alternative basic amino acid, a polylysine tag. Furthermore, when polyionic HI VLPs were constructed with a polyarginine replacement for the native amino acids in the HI loop, and the peptide antigen was tagged with polyglutamic acids, the vaccine failed to induce an immune response. Particle charge could influence other aspects of immunogenicity. As the charged properties of the VLP and antigen are necessary for the linkage reaction, direct comparison of the immunogenicity of a charged and neutral bovine VLP platform is not feasible. 

### 12.5. Transcriptional Profile of Papillomavirus VLP-Treated Dendritic Cells

Dendritic cells are central to the induction of an immune response. To gain further mechanistic insight into the Th1-biased response induced by papillomavirus VLPs, as described above, the transcriptional profile of HPV 16 VLP-treated BMDC was studied by Yang et al. [74]. IFNα/β transcripts were transiently upregulated, whereas the IFN-induced transcripts, CXCL10, and interferon-induced protein with tetracopeptide repeats (IFIT) showed delayed and persistent upregulation. Several cytokines and chemokines were upregulated: IL-1β most prominently, followed by macrophage inflammatory protein 2 (MIP-2), CCL5, CCL7, CCL8, CCL12, and CXCL5. One highly upregulated gene was lymphotactin or XCL1. XCL1 and its receptor XCR1 are involved in cross-priming, and XCR1 is exclusively expressed in conventional dendritic cells [75]. 

### 12.6. Papillomavirus VLP Treatment of Dendritic Cell Subsets 

The nomenclature for dendritic cells has evolved over the decades, and recent work has identified common human and mouse DC subsets designated cDC1 and cDC2 [76]. Mouse cDC1 cells correspond to the older CD8α+ DC, and cDC2 cells to CD8α-/CD11b+ DC. Initially, the significance of mouse DC subsets for the human immune system was unclear because human DCs do not express CD8α. When mouse CD8α+ DCs were shown to express the dead cell receptor Clec9A, also known as DNGR1, and the chemokine receptor XCR1, the finding provided shared markers in the murine and human immune system [77,78]. This work led to the identification of human cDC1 and cDC2 subsets, as these DC cells were previously known to express CD141 (cDC1) and CD1c (cDC2) [76,79]. The resolution of these controversies over nomenclature is beyond academic interest alone because it allows studies in mice to have more direct relevance for humans. The cDC1 subset has antigen handling specializations that distinguish them from cCD2 cells. Specifically, cDC1 cells are much more efficient at cross-presenting cell-bound or soluble antigens on MHC class I [78,80,81,82]. The cDC1 subset produces a unique pattern of cytokines on activation and, of particular importance, is their capacity to produce high levels of bioactive IL-12p70. These cells have been shown to play a key role in viral immunity and in CD8+ T cell responses to noncytolytic viruses and intracellular bacteria [83,84]. The priming of tissue-resident memory T cells has recently been shown to depend on cross-priming by DNGR-1 positive CD8α+ DC in lymphoid organs or non-lymphoid tissues (CD103 + DC) [8]. Tissue-resident memory T cells occupy tissues without recirculating and provide the first response to infectious agents, accelerating pathogen clearance [10,11,85,86]. Tissue-resident T cells have also been recognized to play an important role in tumor immunity [87,88]. As a pure, protein-based vaccine, polyionic VLPs induce CD8+ T cells exclusively by cross priming. The hypothesis that the vaccines generate tissue-resident memory T cells derives from the work, cited above, showing cross-priming by DNGR-1 positive CD8α+ DC inducing T_RM_ cells. However, additional studies are needed to formally demonstrate that polyionic VLP vaccines induce T_RM_ cells. 

The response of HPV 16 VLPs to DC subsets was investigated by us in collaboration with Richard Roden’s laboratory [89]. VLPs were found to bind and enter CD8α+ (cDC1) and CD8α- (cDC2) cells, but the gene expression profile of the subsets was markedly different. VLPs upregulate IFN-α and Th2-related cytokines and chemokines in cDC2 cells and IFN-γ and Th1-related cytokines and chemokines in cDC1 cells. CCR7 was the most highly upregulated gene in cDC1 cells. CCR7 directs the migration of antigen-loaded DCs to the lymph nodes [90]. VLP-treated cDC1 cells upregulated the expression of IL-12b, which is central to Th1 responses. In contrast, VLP-treated cDC2 cells failed to upregulate IL-12b. 

**Figure 12:**
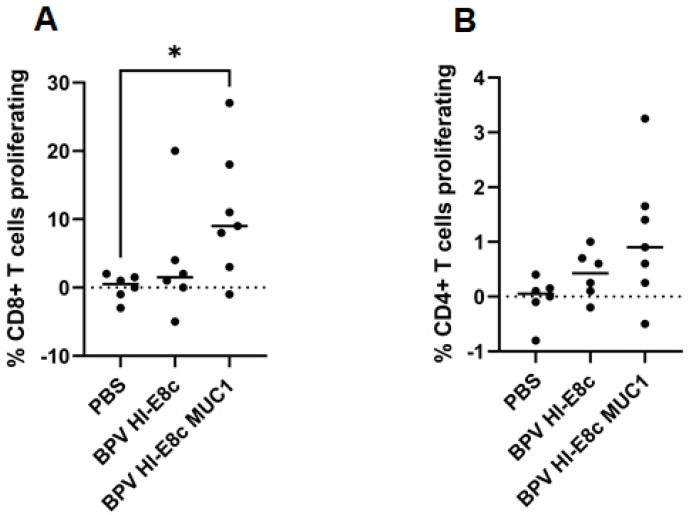
T cell response to MUC-1 polyionic VLP vaccination. MUC1-transgenic mice were immunized subcutaneously three times, 2 weeks apart with 5 μg per dose with vector alone (BPV-HI-E8c), polyionic VLP vaccine (BPV-HI-E8c-MUC1), or PBS (controls). Eleven days post vaccination, mice (6–7 per group) were euthanized, and splenocytes were CFSE-labeled and cultured in the presence of vaccine antigen. Proliferation of MUC1-specific CD8+ T cells (**A**) and CD4+ T cells (**B**) was measured by CFSE dilution using flow cytometry. The difference in CD8+ T cell proliferation was statistically significant (* *p* < 0.05) for BPV HI-E8c MUC1 compared to PBS control. Reprinted with permission from Ref. [12] Copyright 2010 Springer Nature.

CD8+ dendritic cells comprise multiple subtypes. The ability of papillomavirus VLPs to bind and enter and induce a Th1-like gene expression profile in cDC1 cells is likely important for vaccine-induced immunogenicity. Thus, the preferential efficacy of polyionic VLPs for induction of CD8+ T cell responses compared to CD4+ T cell responses may be due to uptake and processing by cDC1 cells [12]. MUC transgenic mice were immunized subcutaneously three times 2 weeks apart with a MUC-conjugated polyionic VLP vaccine (BPV-HI-E8c-MUC), unconjugated polyionic VLPs (BPV-HI-E8c), or PBS (control). A significant increase in the proliferation of CD8+ T cells after in vitro re-stimulation was seen in MUC1 transgenic mice treated with MUC1 conjugated polyionic VLPs (~10%) compared to treatment with PBS (~1%) or vector control (~4%). The proliferative response of CD4+ T cells was >10-fold lower (~0.75%) than that of CD8+ T cells (Figure 12). The 10-fold higher CD8+ T cell response highlighted the ability of polyionic VLP to promote CD8+ T cell responses, unlike most protein-based vaccines that preferentially induced CD4+ T cells.

### 12.7. CD8+ T Cell Response to Polyionic VLPs in Gene-Deficient Mice

The mechanistic basis for the inherent adjuvant activity of polyionic VLPs is uncertain. The activation of TLR signaling is crucial for induction of antigen-specific adaptive immune responses by promoting maturation and activation of dendritic cells [91]. The expression of TLRs is known to differ among DC subsets [92,93]. 

**Figure 13:**
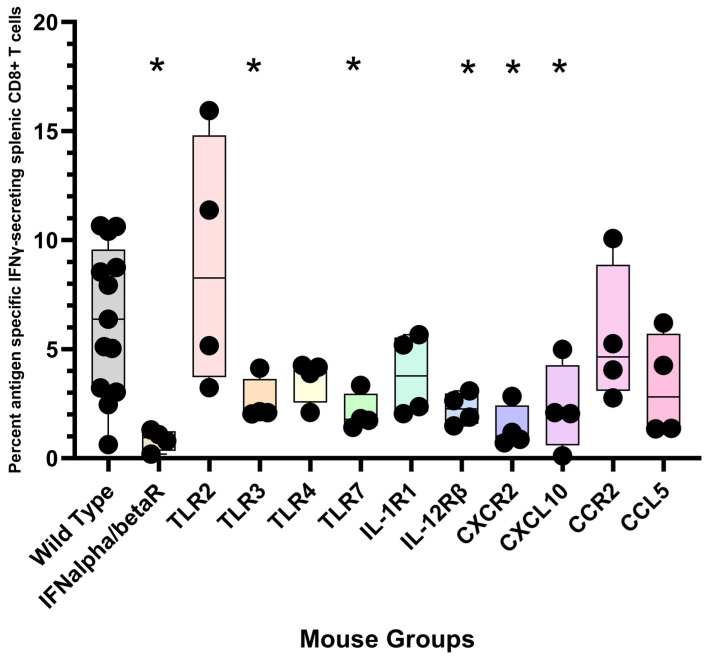
Immunization of gene-deficient mice with polyionic VLPs. Wild-type C57BL6 mice (*n* = 13 mice) and gene-deficient mice (group sizes, *n* = 4 mice) were immunized intradermally, weekly three times, with 20 μg of polyionic VLPs formulated with polyarginine cysteine tagged stimulator of prostatic adenocarcinoma-specific T-cells-1 (SPAS) peptide. Frequencies of antigen-specific CD8 + splenocytes 14 days after vaccination were determined by intracellular cytokine flow assay. Data are displayed as described in the legend to Figure 4. Compared to wild-type mice, CD8+ T cell responses were significantly lower at the *p* < 0.05 level (one tailed Mann–Whitney test) for gene-deficient IFNα/β receptor, TLR3, TLR7, IL-12 receptorβ, and CXCR2 and CXCL10 mice. Groups significantly different from wild-type mice are marked with an “*”.

To investigate which TLR is critical for polyionic VLP-induced CD8+ T cell responses, we immunized mice genetically deficient in TLR2, TLR3, TLR4, and TLR7. TLR3- and TLR7-deficient mice were impaired in the ability to generate an antigen-specific CD8+ T cell response, whereas lack of TLR4 and TLR2 had no effect (Figure 13). For viral immunity, the generation of CD8+ T cell immunity has been shown to depend on TLR 3 signaling [94]. TLR 7/8 agonists have been used as vaccine adjuvants [95], and they have been shown to cross-prime CD8+ T cell responses by the recruitment and activation of cDC1 cells through a Type I IFN and IL-12 codependent mechanism [96]. These results show that signaling through TRL3 and TLR7 is critical for a polyionic VLP-induced CD8+ T cell response. The finding also supports the importance of cDC1 cells since they are the principal DC subset that expresses TLR3 [97,98]. The generation of a CD8 T cell response was also dependent on IFNα/β receptor and the IL-12β1 receptor. The expansion of antigen-specific CD8+ T cells that occurs in response to viral infection is critically dependent on the direct action of type I interferons on CD8+ T cells [99]. IL-12 is known to play an important role in the generation of CD8+ T cell responses [100,101]. Importantly, IL-12 and type I IFN have been shown to cooperatively promote the proliferation of CD8+ T cells [102]. The impaired generation of CD8+ T cells responses in IFN type I and IL-12 receptor knock out mice is consistent with the observation that IFNα and IL-12 are key cytokines upregulated by papillomavirus VLP-treated dendritic cells, as noted above. Additional gene-deficient mice that mounted an impaired CD8+ T cell response included CXCL10 and CXCR2 knock out mice (Figure 13). CXCR3, the receptor for CXCL10, plays a role in the migration of T cells to peripheral tissues and to the lymphoid compartment, where it facilitates the interaction of T cells with APCs leading to the generation of effector and memory T cells [103]. CXCR2 is found primarily on neutrophils, making its role in the vaccine response unclear.

## 13. Conclusions

Despite many successes, the history of vaccinology is also characterized by notable unmet challenges, such as the failure despite many years of effort to develop successful vaccines for malaria, tuberculosis, HIV/AIDS, universal influenza coverage, and cancer. Most vaccines are known or believed to work through the induction of neutralizing antibodies, and research to develop new and improved vaccines has largely focused on new methods to generate such humoral immunity. T cell vaccines are not a modality generally considered for vaccines against infectious diseases despite that cellular immunity is acknowledged to play an important helper role in the generation of antibody responses as well as a role in protective immunity due to the ability of T cells to clear virally infected cells. In this regard, human observational studies have recently shown that T cell responses targeting cross-reactive T cell epitopes can provide complete protection against SARS-CoV-2 by causing an abortive infection [104,105]. 

Whether T cell vaccines will protect against SARS-CoV-2 or other human infectious diseases is unknown because it is a largely untried concept in clinical trials. T cell vaccines have been developed for cancer, where the need for T cell immunity is more self-evident, but most clinical trials have been performed in late-stage cancers after the failure of other treatments. Such an approach is fraught with problems, with the most prominent being a state of systemic or tumor microenvironment immunodeficiency in these cancer patients. Polyionic VLP vaccines have shown efficacy in late-stage cancer in the TRAMP model, as discussed above, raising the possibility that they may be more efficacious in treating advanced stage cancer than other vaccine technologies. The translation of this finding to human cancers awaits clinical trials. 

An additional challenge for the successful development of T cell vaccines is the lack of a clearly defined correlate of protection to guide the immunological goal of vaccine development. The field has relied almost exclusively on measurement of the frequency of antigen-specific cells in peripheral blood. However, recent research in T cell immunology raises the possibility that protective immunity against infectious agents and cancer may depend on induction of tissue-resident memory T cells [85,86,88,106,107,108] and that antigen presentation through cross-priming is the most efficient way to induce these cells [8]. Additionally, the site of antigen delivery may be critical for the induction of robust tissue-resident T cell responses [109,110,111]. Should these discoveries prove foundational, there is a need for new technologies that exploit the cross-priming pathway. Polyionic bovine papillomavirus VLP vaccines are a platform technology with enhanced ability to induce CD8+ T cell responses through cross-priming and thus may fulfill this need. The enhancement for cross-priming is achieved by the immunostimulatory and self adjuvanting properties of papillomavirus VLPs produced in insect cells, together with the rapid transport of antigen to the cytosol by way of the reversible linkage of a polyarginine tagged antigen to the VLP. A formal demonstration that polyionic VLP vaccines induce T_RM_ cells remains to be established, but the prostate cancer vaccine was shown to significantly increase CD8+ T cell numbers in tumor tissues. 

Vaccines are traditionally evaluated in animal models for efficacy prior to testing in human clinical trials. This procedure has worked remarkably well for vaccines that induce neutralizing antibodies that can block the entry of infectious agents when at sufficient levels. In general, efficacy in animal models has had good predictive value for efficacy in human clinical trials of vaccines that induce antibody responses. However, testing the efficacy of T cell vaccines in animal models is problematic. In part, the problem is that induction of circulating T cells in blood, a commonly used readout for immunogenicity, may not be predictive for the possibly more physiologically relevant goal of generating tissue-resident T cells in humans. Additionally, the inherently cross-reactive quality of T cell responses due to the limited repertoire of T cell receptors is of importance [112]. Pre-existing cross-reactive T cells have been shown to affect the response to an immunizing antigen [113,114]. Experimental animals cannot model the exposure history of humans and thus the population of pre-existing memory T cells in humans [115,116]. This limits the predictive value of animal models for the performance of T cell vaccines in clinical trials. While testing T cell vaccines in animal models is a necessary first step in vaccine development, exploring the true potential of a T cell vaccine to prevent or treat human diseases will require a degree of flexibility in the decision to advance novel technologies into clinical trials. This admonition applies to polyionic VLP vaccines, as well as other T cell vaccine technologies.

## Figures and Tables

**Table 1:** Comparative efficacy of vaccines in the TRAMP mouse model of prostate cancer.

Vaccine	Age at Administration	Measure of Tumor Burden	Efficacy	Comment	Reference
Dendritic cells pulsed with tumor cells	8 wks	Genitourinary tract weight to whole body weight ratio	33%		[49]
STEAP Simian adenovirus prime and vaccinia boost	6–8 wks prime 7–11 wks boost	Genitourinary tract weight to whole body weight ratio	20%		[22]
Tumor cell lysate + anti-CTLA4	14 wks	Prostate weight	0%	Incidence of tumors was reduced by 26%	[43]
PSCA and STEAP DNA prime and vaccinia boost	7 wks prime 11 wks boost	Genitourinary tract weight	40%		[50]
Tumor cell lysate + CpG adjuvant in microspheres	Repeated at 10, 12, 14, and 16 wks	Prostate tumor volume by magnetic resonance imaging	80%	Adjuvanted microspheres alone had 60% efficacy	[51]
PSCA DNA prime and VEE self-replicating mRNA boost	8–10 wks prime 10–12 wks boost	Survival at 200 days	20%	80% efficacy at 1 year	[23]
PSCA, SPAS-1 and PAP Polyionic VLP vaccine	19–20 wks	Prostate weight	43%		[21]
PSCA, SPAS-1 and PAP Polyionic VLPs + anti-PD1	19–20 wks	Prostate weight	63%		[21]

## Data Availability

Not applicable.
